# Ecology of the Anthropocene signals hope for consciously managing the planetary ecosystem

**DOI:** 10.1073/pnas.2024150118

**Published:** 2021-07-09

**Authors:** Clarence Lehman, Shelby Loberg, Michael Wilson, Eville Gorham

**Affiliations:** ^a^College of Biological Sciences, University of Minnesota, Saint Paul, MN 55108;; ^b^Department of Ecology, Evolution, and Behavior, University of Minnesota, Saint Paul, MN 55108;; ^c^Institute on the Environment, University of Minnesota, Saint Paul, MN 55108;; ^d^Division of Science and Mathematics, University of Minnesota, Morris, MN 56267;; ^e^Department of Anthropology, University of Minnesota, Minneapolis, MN 55455

**Keywords:** demographic transition, logistic and orthologistic growth, anthropology, sustainability, possibilist agenda

## Abstract

Human populations have grown to such an extent that our species has become a dominant force on the planet, prompting geologists to begin applying the term Anthropocene to recognize the present moment. Many approaches seek to explain the past and future of human population growth, in the form of narratives and models. Some of the most influential models have parameters that cannot be precisely known but are estimated by expert opinion. Here we apply a unified model of ecology to provide a macroscale summary of the net effects of many microscale processes, using a minimal set of parameters that can be known. Our models match estimates of historic and prehistoric global human population numbers and provide predictions that correspond to some of the more complicated current models. In addition to fitting the data well they reveal that, amidst enormous complexity in our human and prehuman past, three key ecological discontinuities have occurred in turn: 1) becoming dominant competitors of large predators rather than their prey, 2) becoming mutualists with food species rather than acting as predators upon them, and 3) changing from a regime of uncontrolled population growth to one of controlled fertility instead. All three processes have been interlinked with cultural evolution and all three ushered in developments of the Anthropocene. Understanding the trajectories that have delivered us to this stage can help guide prudent paths into the future.

## Significance

Human populations so dominate the ecology of the planet that geologists are describing this as the Anthropocene, “the time of humans.” Therefore, understanding the ecology of our time requires understanding our growth, which multiple models have sought to explain. Here we apply a unified model of ecology to understand and summarize historic and prehistoric human populations and provide predictions that concur with some of the more complicated current methods. They reveal that in our societies, and in those of our prehuman ancestors, changes involving cultural evolution have altered fundamental ecological mechanisms, producing three great ecological discontinuities that are successively related to tools, agriculture, and control of fertility. Understanding our ecological development thus far can help guide us into our future.

Recent times have been called the Anthropocene (1, 2) to acknowledge a profound human signature on the planet. Determining what mechanisms are responsible therefore is central to the discussion (3). The ecology of hominins—the human branch on the tree of life—provides clues to such mechanisms.

**Fig. 1.**
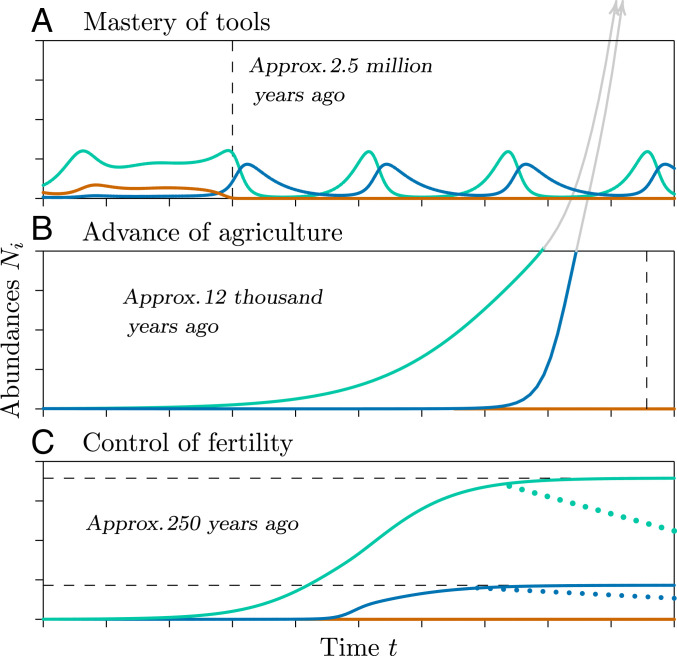
Dynamics of the three major stages in human ecology, enabled by cultural change. Representative predator abundance is shown in red, prey abundance in green, and hominin abundance in blue. All solid curves are numerical solutions to the ecological equation 1 in Fig. 2, using parameters of *SI Appendix*, section S1. (*A*) The hunter-gatherer phase, beginning in the remote past when parameter $s_{1,2}$ in equation 1 in Fig. 2 became negative. Top predators in red were engaged by weapons-wielding hominins (left of dashed line), top predators declined to low levels, and hominins rose to dominate the food web, illustrated here with predator–prey cycling (right of dashed line). Such cycling is one of multiple possible outcomes from predator–prey interactions, which can also include equilibrium, extinction, and chaotic dynamics. (*B*) Agricultural phase, beginning when parameter $s_{3,2}$ became positive, creating a form of mutualism with agricultural animals and crops. The dashed vertical line marks a singularity—a time before which population growth is guaranteed to be arrested by some ecological, social, or physical limitation. Advances in health contribute to the latter parts of this curve, although the runaway nature of the curve appears to have been invariant for millennia. (*C*) Contemporary phase, when moderation of human fertility arose in recent centuries as parameter $s_{2,2}$ declined. The dashed horizontal lines mark hypothetical carrying capacities—approximate populations where growth rates approach zero, according to equation 1 in Fig. 2, even with mutualistic interactions intact. The dotted lines represent possible planned or unplanned reductions in global population to unknown levels, as suggested by Fig. 4, indicating fertilities falling below replacement.

Whether the official beginning of the Anthropocene is marked by incipient agriculture or the nuclear era, changes leading to it were driven by cultural and ecological interactions that are evident in basic multispecies equations of population growth. Changes in the processes modeled by those equations have precipitated sharp ecological discontinuities in human population growth (Fig. 1 *A*–*C*), which correspond to changes in sign among paired parameters in the equations. Between the ecological discontinuities, amidst all of the complexity of social and technological development, overall human population numbers have steadfastly mirrored solutions of the basic equations.

## Modeling Populations.

Human population growth is a key factor in the Anthropocene, but modeling human population growth has presented successive difficulties.

Following Malthus (6), attempts to predict human population growth have presumed that human populations will face limits to their growth—that is, ecological carrying capacities. Given that Earth is finite, all ever-expanding biological populations—plants, humans, and other animals—ultimately face an inevitable depletion of space, resources, or other limiting factors. Verhulst (7) modeled this expectation with a now-celebrated form that he named the “logistic equation”. In the 20th century, Pearl (8) and others assumed that human populations always followed a logistic growth pattern, even though the data available then did not fit that form. Pearl (8) and others predicted a succession of carrying capacities for humanity, but global populations surpassed each successive prediction without pause (9). Thus, while models imposing a predictable carrying capacity on human populations have largely seemed reasonable, they have largely been wrong.

More than 30 y after Pearl (8), von Foerster et al. (10) fitted human population estimates for the past two millennia without the constraining assumptions of a carrying capacity and found they fitted a kind of hyperbolic equation that kept doubling at ever increasing rates. This led them to predict that the population would exceed all bounds at some finite time in the future (10). This was in exact opposition to the dynamics so long assumed by Pearl (8) and others and was met with criticism and disbelief (11). Twenty-five years after that, Cohen (9) exhibited a model that retained a carrying capacity, but that let the carrying capacity systematically increase faster than the population increased. That could also mimic runaway human populations in ways that fit the actual data.

Some groups working with detailed census data do not attempt to formulate a general model for projecting populations, but instead calculate a new population value for each geographic region by taking the present population of a region, adding expected births and immigration while subtracting expected deaths and emigration during an upcoming time interval, in what is described as a “straightforward bookkeeping procedure” (12). Projections are developed with expert opinion, which is said to benefit from a lower data requirement than curve fitting (13), and augmented with “ex postfacto analysis” (13, 14), wherein observed errors in previous estimates become adjustment factors to help correct unknown errors in present estimates. This approach is useful in forecasting populations over some timescales, but involves increasing uncertainty over long timescales.

**Fig. 2.**
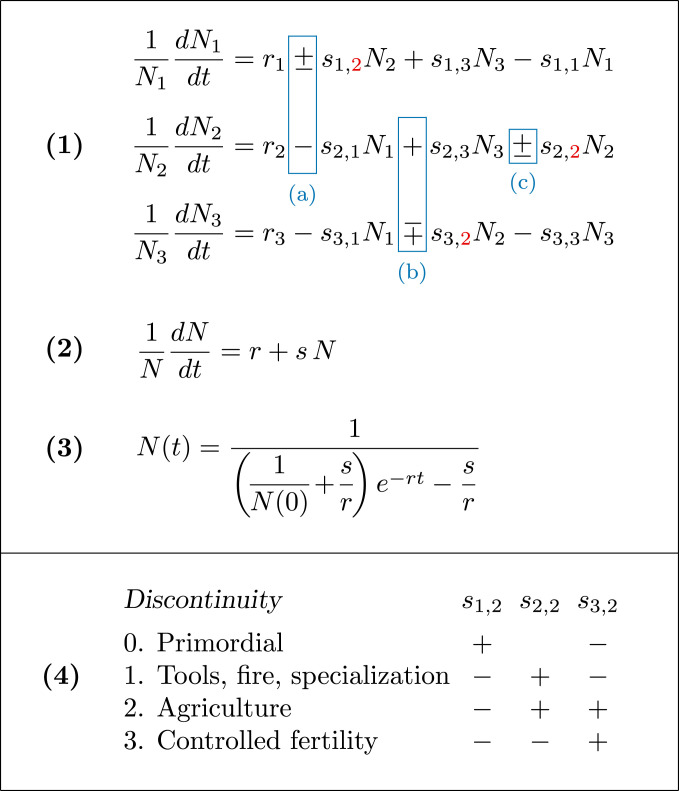
Ecological dynamics. Here the r parameters represent density-independent growth rates that dominate at low population levels, while the s parameters are largely ecological and social, representing interactions between and within species. $N_{1}$ symbolizes the abundance of predators of hominins and other prey, $N_{2}$ symbolizes hominin abundance, and $N_{3}$ symbolizes the abundance of species eaten by hominins. (*1*) Coupled equations representing three trophic levels. Boxed in blue are the three relationships that were under control by us or our hominin ancestors and that changed to cause the discontinuities, (*a*) from predation (+,−) to competition (−,−), causing the first discontinuity, leading to Fig. 1*A*; (*b*) from predation (+,−) to mutualism (+,+), causing the second discontinuity, leading to Fig. 1*B*; and (*c*) from orthologistic (+) to logistic (−), causing the third discontinuity, leading to Fig. 1*C*. A generic procedure to solve these coupled equations and exhibit the dynamics of Fig. 1 appears in *SI Appendix*, section S2. (*2*) Reduced dynamics of conditions after the second discontinuity. It can be shown that for mutualists interacting according to the equations for $N_{2}$ and $N_{3}$, when the mutual enhancement terms $s_{i,j}$ overpower the self-interaction terms $s_{i,i}$, the species move toward fixed ratios and then the multiple equations describing their growth collapse to this single equation (4). This is a first-order approximation of the general power-series expansion of Hutchinson for population growth (5), which suffices for long-term human dynamics. (*3*) Explicit solution to equation 2, used to fit population data. (*4*) Summary of changes in sign causing the discontinuities. The sign of the self-limiting term $s_{2,2}$ is unclear during the primordial phase. Signs of parameters not shown, such as $s_{2,1}$, do not necessarily change; for example, predators would still pose risks to hominins when present, but the overall effect would become negligible as predators were reduced to low levels in areas where hominins resided.

**Fig. 3.**
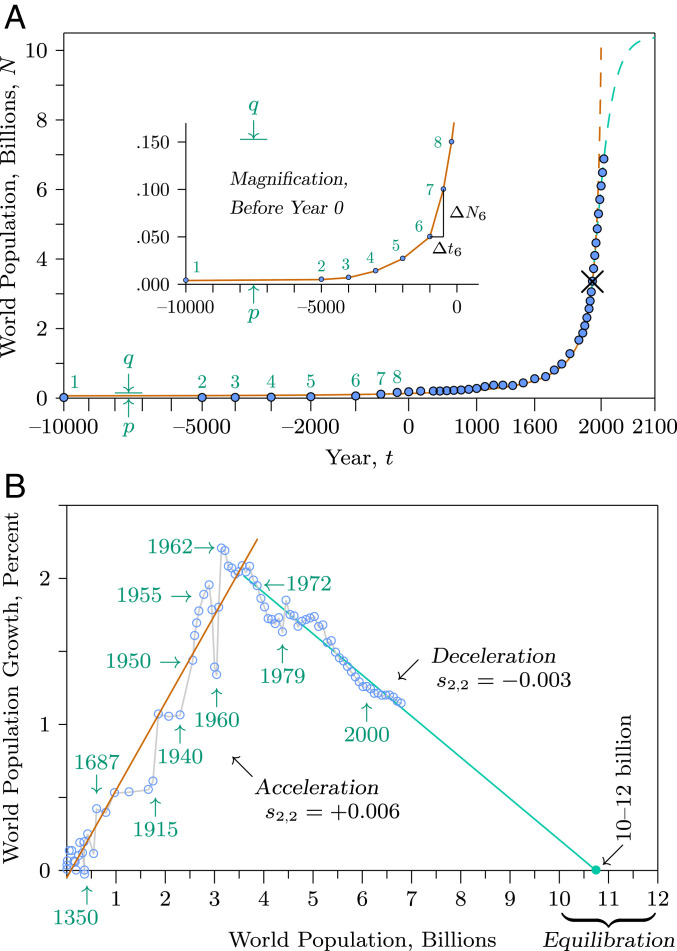
World population data. (*A*) Blue dots show estimated world population over time (*SI Appendix*, section S3). The red curve is equation 3 in Fig. 2 fitted to the data from 10,000 BCE to the 1960s AD, characterized by accelerating growth. The green curve is equation 3 in Fig. 2 fitted to the data since the discontinuity in the mid1960s, characterized by decelerating growth. The × marks the discontinuity. Arrows labeled p and q in the main graph are stretched vertically to matching arrows marked p and q in *Inset*. Points labeled 1 to 8 in the main graph are the same as points 1 to 8 in *Inset*. (*B*) Per capita growth of the population as a percentage plotted against the population itself. The vertical axis is calculated as $1/N_{i}\;\Delta N_{i}/\Delta t_{i}$, between successive population points, as depicted for $\Delta N_{6}$ and $\Delta t_{6}$ in *A*, *Inset*. The light gray lines form a timeline connecting successive data points. The upward-sloping red line fits equation 3 in Fig. 2 to the data before the discontinuity marked by × in *A*, representing a 12,000-y-long period of rapid orthologistic growth. The downward-sloping green line fits the data following the discontinuity marked by the × and projects the data forward to a hypothetical equilibrium. The ecological parameter $s_{2,2}$ shown for both the red-line acceleration phase and the green-line deceleration phase represents human population growth interacting with mutualistic plants and animals and with continuing cultural development, reaching equilibration as the percentage growth approaches zero. Note how abruptly the global average slope indicated by parameter $s_{2,2}$ changed from positive to negative at the time of the third discontinuity.

All of the methods above are fragmentary in that none can explain the full range of human population dynamics. Pearl (8) assumed that the world’s population at his time must be decelerating toward a carrying capacity, even though it was increasing at an accelerating rate in terms of percentage of growth, which would become evident if his data were plotted in a different form (see Fig. 3*B*). The method used by von Foerster et al. (10) worked for populations up to the time of his observations, but was ad hoc in its formulation—simply an ever-accelerating curve that fitted the data. Moreover, that model had no way for the population to break away from its ever-accelerating path, as eventually it must do on a finite planet. The method explained by Cohen (9) also can fit that range of data, but can break away from its ever-accelerating path only by imposing an external carrying capacity that eventually limits the population. Finally, the use of expert opinion with ex postfacto analysis does little to explain or predict novel possibilities. Have we overshot our ecological carrying capacity, are we approaching it, or are we well under it as new technological developments might allow? To understand the ecology of the Anthropocene, the present analysis applies something different—a single unified model of ecology (4) that accommodates what we and our prehuman ancestors have experienced from our beginning until today (Figs. 1 and 2), to make sense of our past and to examine our possible future.

## Dynamics Shaping the Anthropocene.

To expand beyond basic models, human population dynamics can be cast in relation to such features as land cover and technological advancement (15), but interactions with other species in the food web—through ecological cooperation, competition, and exploitation—provide an even more complete picture. These interactions can be represented as a three-dimensional unified system of ecological equations (equation 1 in Fig. 2), a general form explored by Volterra (16) and used here to illustrate motifs of basic ecological interactions (4). In certain cases these equations reduce to one-dimensional form (equations 2 and 3 in Fig. 2). When parameter s is negative, the one-dimensional form is equivalent to the textbook logistic growth equation, and when s is precisely zero, it represents exponential growth. When s is positive, the equation represents orthologistic growth (4), a form that characterizes populations in rapid growth phases.

In three-dimensional form, $N_{1}$ symbolizes the abundance of top predators, $N_{2}$ symbolizes hominin abundance, and $N_{3}$ symbolizes prey abundance, which can include animals hunted and plants gathered by hominins. Individual parameters of the form $s_{i,j}$ measure how increased abundance of one species affects population growth of the other. For example, parameter $s_{1,2}$ represents the effect that the abundance of species 2 has on the growth rate of species 1, and $s_{2,2}$ represents the effect of species 2 on itself. In general, matched pairs of parameters, $s_{i,j}$ and $s_{j,i}$, categorize the ecological interactions.

## Interactions with Predators.

The first great ecological discontinuity in population dynamics involved our early hominin ancestors and their predators. Rapidly in evolutionary time, as weapons and social organizations advanced (17), predators that may have long benefited from hominins as prey instead were harmed by their presence. That is, the $s_{1,2},s_{2,1}$ pair changed from plus–minus, which is predation, to minus–minus, which represents competition. With tools and fire, our ancestors outcompeted their predators and eventually dominated the food web (18). But the relationship between our early ancestors and their prey or other food—the $s_{2,3},s_{3,2}$ pair of parameters—remained plus–minus. A prolonged hunter-gatherer phase persisted over 2 million y. Our early ancestors experienced periodic population growth and decline (19), complex as in Fig. 1*A* or more complex than that. Even during early development of agriculture, long-term population growth appears to have been slow, with periods of rapid growth punctuated by episodic reductions (20).

## Interactions with Prey.

The second great ecological discontinuity began with a seemingly innocuous transformation when our now-human ancestors unconsciously changed the sign of the parameter pair $s_{2,3},s_{3,2}$ from plus–minus to plus–plus (Fig. 1*B*). This mere change in sign would have involved fundamental changes in cultural evolution, from the dispersed resources of a forager economy to practices such as fixed property rights (21). Then, through intentional protection and cultivation of plants and animals that were formerly prey, agriculture increased the carrying capacity of the cultivated species, which in turn increased human carrying capacity, forming a positive feedback loop. As others have noted (22), agriculture is a mutualism. Although it may seem dubious to consider domesticated plants and animals that we kill and eat as mutualists, they meet the mathematical requirements of mutualism with both interaction terms positive (Fig. 2). Both populations can expand so rapidly that, unchecked, they would soon exceed all bounds, in what is called a finite-time singularity. This is related to what arose earlier in fitting the rapid growth of human populations (9, 10), but here it results from known ecological interactions.

The presence of a singularity may make it seem that the equations need terms of higher order than shown in Fig. 2, to remove the singularity (23). However, all mutualisms are embedded in larger food webs. Natural mutualisms increase only until they are checked by some other natural force (24), such as predation, disease, or resource depletion. In contrast with natural mutualisms, our ancestors kept their mutualisms pure, suppressing forces that check growth. They hunted and killed predators of domesticated animals, fertilized crops, weeded them to eliminate plant competitors, and fenced them to exclude herbivores. Keeping mutualisms pure produced a signature of runaway growth as populations repeatedly broke through postulated limits (Fig. 3*A*).

There are uncertainties in details. The runaway growth could have been accompanied by increased local mortality and morbidity, at least in early stages. Ecological growth is maintained in the equations by the combination of the intrinsic growth rate r and the social interaction term s, with organic and cultural evolution affecting each term. Early agriculture could increase the social term s and hence could induce growth in larger populations even if it reduced the intrinsic growth rate r and caused distress in smaller populations. Coupled evolution of r and s can be expected, but could be difficult definitively to detect with limited data available from ancient times.

The dramatic increase in total human population in the past few centuries may seem like a recent explosion, characterized by a long flat portion for most of history followed by a rapid upturn, commonly described as the shape of a hockey stick. However, examination of the flat portion reveals that it is not flat at all—it also swings upward, with much of it still looking flat (Fig. 3 *A*, *Inset*). That is the nature of rapidly increasing growth. Thus, the present upturn is a continuation of a much longer trend. Frequent disturbances by war and disease, and even collapse of local populations, caused serious lags and reversals, but did not stop the overall increase in orthologistic growth.

When species rapidly increase due to sustained mutualistic interactions, the species abundances in the multidimensional form of equation 1 in Fig. 2 approach fixed proportions and that equation collapses to the single-dimensional form of equation 2 in Fig. 2, where the mutualists jointly can be represented by different proportions in a single equation (4). Therefore, human population growth curves as in Fig. 3 represent just the human portion of a multispecies mutualism. Indeed, the human species does not now dominate the Earth. The mutualisms dominate (25). Rice, wheat, corn, cattle, chickens, and other domesticated species are now, with humans, the most abundant large organisms on Earth (26).

Recently our species outgrew the need for animal mutualists to power plows and locomotion and also for long-established renewable-energy systems such as sailing ships and water wheels, substituting engines consuming fossil fuels instead. We also outgrew the need for animals to fertilize crops, substituting artificial nitrogen fertilizer created in the Haber–Bosch process. The need for animals and plants for clothing diminished with synthetic cloth and synthetic furs. All these brought us to a partially nonbiological world, still developing, where not even the carrying capacities of our living mutualists need be limiting. Still, a large portion of the land surface is dedicated to growing food for ourselves and our mutualists, which has displaced wild species and induced a large extinction debt (27, 28). Can such an extinction debt be repaid with developments of the future, such as tissue-culture food (29) grown on nutrients extracted from diverse natural plant materials in restored ecosystems?

## Interactions within.

In human populations benefiting from cooperation and specialization, $s_{2,2}$ was positive. The more people in a society, the more specialists, inventors, and researchers there could be (9), leading to feedbacks for ever-higher per capita growth rates. But starting in the 18th century and spreading with the industrial revolution, the modern demographic transition had its early beginnings. In specific regions, occurring at different times in different places, death rates slowly dropped, thereby increasing population growth, and after a time lag of decades or more, birth rates subsequently dropped, tending to restore a former growth rate (30).

Fig. 3*B* illustrates increasing per capita growth along the red upward-sloping trend line, averaged across the globe. The individual points and the gray lines connecting them in sequence show deviations from the trend. The global plague marked with the year 1350 shows a massive decline in the worldwide rate of growth, indeed to negative values with world population diminishing. That is also perceptible in Fig. 3*A*. However, much detail that is obscured in Fig. 3*A* is revealed clearly in Fig. 3*B*. Deviations below the red trend line are visible when deaths increased, as during the plagues of the past but also during the two 20th-century world wars, marked with 1915 and 1940. A globally averaged surge above the red 12,000-y trend line appears halfway through the 20th century, as global wars ameliorated (31) and advances spanning technology, medicine, science, and sanitation accumulated (32, 33). Mortality markedly fell while birth rates remained relatively unchanged, inflating per capita growth on the global scale, as marked in years 1955 and 1962. This was interrupted by tragic regional mortality related to the Great Chinese Famine surrounding 1960.

Then, in the mid1960s, the third great ecological discontinuity arose, completing the second phase of the modern demographic transition and unexpectedly ending 12 millennia of rapid acceleration. However, unlike standard views of demographic transitions (30), which presume populations will return to their previous rates of growth, birth rates did not fall until they compensated for reduced death rates, but began a long-term continuing decline that has now spanned over half a century—marked by the green downward-sloping long-term trend line in Fig. 3*B*. And that long-term trend suggests viable paths into the future.

Demographers have proposed multiple causes of transitions to low fertility (30). Here we emphasize recent changes that reflect the timing of the ecological discontinuity in Fig. 3*B*, related to the changing status of women in society. This recent effect corresponds in time to 1) advances in medicine and sanitation becoming widespread, reducing childhood deaths and making frequent births unnecessary; 2) invention of new synthetic methods of birth control at the disposition of women, making less frequent births possible (34); 3) women moving into all aspects of the workforce (35), making frequent births demanding; and 4) increasing importance of education for gaining employment, making delayed reproduction desirable. Ecologically, the numerous factors contributing to the deceleration manifested themselves as a mere change of sign in the self-limiting parameter $s_{2,2}$—from positive to negative or becoming more negative (Fig. 2, part *4*).

**Fig. 4.**
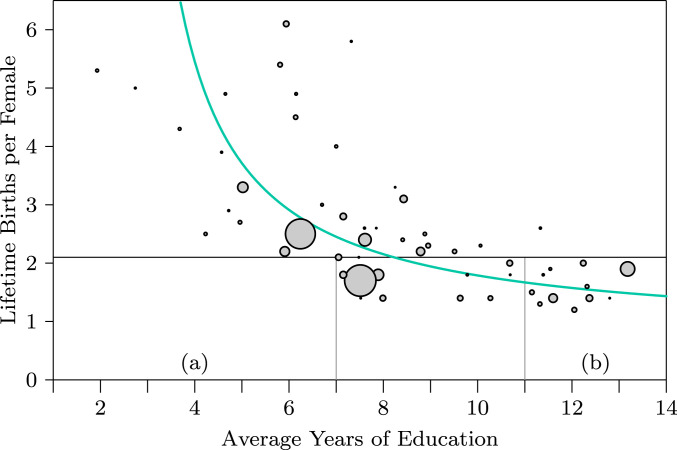
Fertility and education. Fertility tends to decline with increasing years of education (*SI Appendix*, section S4). Each circle represents a nation of 10 million people or more, with the areas of the circles proportional to population size. The horizontal line marks the replacement rate, where the population growth rate is zero. The green curve is a fitted hyperbola to illustrate the pattern. Similar patterns arise in graphs of fertility versus per capita national income, per capita energy use, ratio of female to male education, and other correlates. Of nations with fewer than 7 average years of education, none have fertility below replacement levels (area *a*). With one exception, among all nations with over 11 average years of education, fertility is below replacement (area *b*).

In the 21st century, after only about 50 y of decelerating growth, the global population continues to increase but births in many societies have dropped below replacement levels (Fig. 4), with declines in resident populations compensated in some countries by immigration. The relative rapidity of this change—just half a century—may spill over into social disjunction (36).

Other factors contributed to the deceleration. Increasing attention to consequences of overpopulation (37, 38) and growing consciousness of Earth’s fragility, visually reinforced by images of the whole Earth from space (39), plus myriad other causes (40–44), may also have contributed. Thus, moderation of population growth may have been driven appreciably by local and family-level decisions, where improved public health and decreased childhood mortality enabled families to invest more resources in fewer offspring.

Whether reduced fertility is adaptive—or maladaptive in the sense of decreasing long-term lineage survival—remains in debate (43, 45). What may become important is not genetic fitness measured by the number of offspring in the next generation, but rather the number of offspring surviving to create the nth generation forward. Limited fertility may be a strategy to optimize fitness in a world of low childhood mortality and transfers of wealth (46). Economies driven by intensive investments in cultural capital may have low fertility, but be offset by higher rates of technological innovation as a consequence—leading to increases in wealth in present and subsequent generations and long-term reductions in violence (31).

Assuming the deceleration continues at current rates, the ecological models presented here predict that overall human population size will level out between 10 and 12 billion sometime in the next century (Fig. 3*B*), consistent with other estimates (47). It even appears possible that continued trends toward birth rates lower than replacement rates (Fig. 4) could lead to reductions in world population, lower than the equilibrium levels suggested in Fig. 3*C*.

## Recent Past and Present.

The sustained deceleration phase means that uncontrolled population growth per se is no longer one of humanity’s largest concerns. Rather, the accumulated effects of past centuries of uncontrolled growth, including increasing consumption and emissions per capita, are the subjects of concern. Global temperatures are rising and thawing permafrost, threatening to increase temperatures further still (48); biodiversity appears to be on a path of dramatic decline (49); endocrine disruptors and other chemicals are toxifying the environment (50); floating plastics contaminate the seas (51); emerging diseases threaten stability now and in the future (52); and much more.

Yet within all of the disruptions, we have successfully healed a number of large self-inflicted wounds. Fallout from above-ground nuclear testing is all but gone; human population growth—the population bomb—is being defused; per capita deaths from warfare have been decreasing; sulfuric-acid rain is restored to 19th century levels over large areas; extinction of some species from overhunting, such as whales, partly has been arrested; and the ozone hole has shrunk. These examples are relatively recent, with awareness of our powers emerging at different times in different groups and places, but they show that while an enormous burden remains, indeed it is possible, politically and physically, to heal large self-inflicted wounds.

## The Possibilist Agenda.

Considering what is possible, and cutting on a separate plane across the range of attitudes from cynicism to pessimism to optimism to Pollyannaism, is “possibilism.” In approaching self-inflicted problems of global scale, and also much more local problems, possibilism recognizes that the course of human events largely is not the domain of probability. Probability derives from combinations of many connected steps beyond our influence or knowledge. In human events, outcomes often depend on only a few major steps, often not beyond our influence or knowledge.

Therefore, in following the possibilist agenda, one first evaluates and eliminates what is impossible—what cannot occur by the laws of the universe. What remains is the tentatively possible, including both the desirable and the undesirable. Following the possibilist agenda means working tirelessly to imagine both possibilities and impossibilities and then laying plans to arrange events so that the desirable can be realized and the undesirable avoided—working to avoid unintended consequences. In this way, by superposing such thoughts onto recognized physical–biological–social problems of the world, including those described above, seemingly intractable problems may have possibilist solutions.

## Directions Forward.

Accompanying our domination and disruption of the planet has begun a conscious awareness of the magnitude of our powers to help guide us to prudent paths into the future. Ours is the first species to become aware of our global scope, the first to organize global communication and satellite monitoring of the planet as a whole, and the first consciously to consider how to create a sustainable planet.

We now face our next daunting challenge—learning to manage our role on the planet for our continued existence and—we can hope and expect—for that of our fellow creatures. Will our modern existence be a minor blip on the geological timescale, as indicated by the suffix “-cene” in “Anthropocene”? Or will we be able—as geologist Stoppani (53) suggested a century ago—to elevate our existence to the dignity of an era and advance the Anthropocene into the Anthropozoic?

There is reason for hope. It may seem unimaginable that we can learn to manage consciously the entire planetary ecosystem. We should, however, remember that throughout our relatively short history, the unimaginable repeatedly has morphed into the commonplace.

## Materials and Methods

1) To explore possible population dynamics of hominins, predators, and prey over time (Fig. 1 *A–C*), we applied the equations shown in Fig. 2, part *1*, solving the differential equations numerically with a Euler method over small finite time steps. We present all of the parameters used to create Fig. 1 in *SI Appendix*, section S1. The computer code that we wrote to solve the equations is exhibited in *SI Appendix*, section S2. 2) To analyze global human population dynamics (10,000 BC to present, Fig. 3), we used publicly available population estimates from the US Census Bureau and other sources, tabulated in *SI Appendix*, section S3 with sources cited. We used equation 3 in Fig. 2 in a nonlinear least-squares fit to data points before and after the discontinuity to obtain the curves and lines summarizing the data. 3) To explore the relationship between fertility and education (Fig. 4), we used publicly available data from the World Bank, tabulated in *SI Appendix*, section S4 with sources cited. Those data points are fitted with linear least squares to a hyperbola for illustration in Fig. 4.

## Supplementary Material

Supplementary File

## Data Availability

All study data are included in this article and/or *SI Appendix*.
