# Supplementary material for: Ecology of the Anthropocene signals hope for consciously managing the planetary ecosystem
Source: Proc Natl Acad Sci U S A. 2021 Jul 9;118(28):e2024150118. doi: 10.1073/pnas.2024150118 (PMC8285894; doi:10.1073/pnas.2024150118)
Supplement: Supplementary File [file pnas.2024150118.sapp.pdf]

SUPPORTING INFORMATION

accompanying

ECOLOGY OF THE ANTHROPOCENE SIGNALS HOPE FOR CONSCIOUSLY MANAGING THE PLANETARY ECOSYSTEM

by

Clarence Lehman, Shelby Loberg, Michael Wilson, and Eville Gorham

This document carries supporting information for the paper named above. It allows the figures of that paper to be reproduced, compared with other sources of data, extended into the future as new data become available, or adapted to other purposes.

The data are recorded in the Centinel archival database format (1,2), which is intended for long-term storage of data and metadata as well as reliable processing in computer programs.

Each of the figures in the paper is represented by a separate appendix in Centinel format below. Supporting references follow the last appendix.

```
Appendix S1: Parameters, three phases of population growth

Contents:  Below are the data for Figure 1, recorded in Centinel format (1, 2).

Label Fig:     Part of the figure represented, A-C
Label i:       Species index, 1-3
Label r{i}:    Intrinsic growth rate of species i, i=1-3
Label s{i,j}:  Effect on species j abundance on species i growth, i,j=1=3
Label N{i}:    Initial population of species i, i=1-3
Label Type:    Type of interaction


Table:
```

Fig	i	r{i}	s{i,1}	s{i,2}	s{i,3}	N{i}	Type
A	1	-0.5	0.01	-5.0	0.5	0.45385295	Predator-prey
A	2	-0.5	-1.0	0.01	0.5	0.0915	
A	3	1.0	-1.0	-1.0	-0.01	1.0	
B	1	-0.5	-0.1	-0.5	0.0	0.01	Orthologistic
B	2	-0.5	-1.0	-1.0	1.0	0.01	
B	3	1.0	0.0	0.125	-0.1	0.01	
C	1	-0.5	-0.1	-0.5	0.0	0.01	Logistic
C	2	-0.5	-1.0	-4.0	1.0	0.01	
C	3	1.0	0.0	0.125	-0.1	0.01	

SUPPORTING INFORMATION

Appendix S2: Procedure, solution of population dynamics

Contents: Below is a procedure to solve Equation 1 of Figure 2 in the paper. Any set of 15 parameters, as in Appendix S1 above, defines a three-component motif whose population dynamics can be resolved with differential equation software, including the explicit Euler-method below. For example, the 15 parameters appearing below are extracted from the first three-line set in Appendix S1 above, and generate the data for Figure 1A in the paper.

The procedure below is generic and, properly encapsulated with any required language-dependent variable declarations and function definitions, will run under various procedural programming languages such as R, C, C++, Java, JavaScript, or AWK.

```
r1=-0.5; s11= 0.01; s12=-5.0;  s13= 0.5;  N1=0.45385295;
r2=-0.5; s21=-1.0;  s22= 0.01; s23= 0.5;  N2=0.0915;
r3= 1.0; s31=-1.0;  s32=-1.0;  s33=-0.01; N3=1.0;

dt=.001; pt=.1; p=pt+dt; t=0; tmax=50; Nmax=20; |Establish control variables.

while(t<tmax+dt)                                |Loop through time and
{ p=p+dt; if(p>pt) { output(t,N1,N2,N3); p=0; } |periodically display results.

  dN1 = (r1 +s11*N1 +s12*N2 +s13*N3) *N1*dt;    |Calculate growth of each
  dN2 = (r2 +s21*N1 +s22*N2 +s23*N3) *N2*dt;    |population during the current
  dN3 = (r3 +s31*N1 +s32*N2 +s33*N3) *N3*dt;    |time step.

  N1=N1+dN1; N2=N2+dN2; N3=N3+dN3; t=t+dt;      |Update the population values,
  if(N1<0 or N1>Nmax) break;                    |check them for range, and
  if(N2<0 or N2>Nmax) break;                    |repeat for the next
  if(N3<0 or N3>Nmax) break; }                  |incremental time step.
```

SUPPORTING INFORMATION

Appendix S3: Data, population and percent growth

Contents: Below are the data for Figure 3 in the paper. Three methods, all similar, are calculated for percentage growth, 'Left', 'Mid', and 'Log'. The 'Mid' method is used in the figure. The data are recorded in Centinel format (1, 2).

Label t: Year (negative=BC)
Label N: Population at time t, billions
Label Left: Percent growth, calculated as (N1-N0)/(t1-t0) / N0
Label Mid: Percent growth, calculated as (N1-N0)/(t1-t0) / ((N1+N0)/2)
Label Log: Percent growth, calculated as (log(N1)-log(N0)) / (t1-t0)
Label Ref: Source of t and N (references follow the last appendix)
Label Note: Comment on the data

Table:

t	N	Left	Mid	Log	Ref	Note
-10000	0.004	0.005000	0.004444	0.004463	(3)	Second discontinuity
-5000	0.005	0.040000	0.033333	0.033647	(3)	
-4000	0.007	0.100000	0.066667	0.069315	(3)	
-3000	0.014	0.092857	0.063415	0.065678	(3)	
-2000	0.027	0.085185	0.059740	0.061619	(3)	
-1000	0.050	0.200000	0.133333	0.138629	(3)	
-500	0.100	0.166667	0.133333	0.135155	(3)	
-200	0.150	0.066335	0.062189	0.062270	(3)	
1	0.170	0.059119	0.055835	0.055892	(3)	
200	0.190	0.000000	0.000000	0.000000	(3)	
400	0.190	0.000000	0.000000	0.000000	(3)	Collapse of Rome
500	0.190	0.052632	0.051282	0.051293	(3)	
600	0.200	0.050000	0.048780	0.048790	(3)	
700	0.210	0.047619	0.046512	0.046520	(3)	
800	0.220	0.090909	0.086957	0.087011	(3)	
900	0.240	0.104167	0.099010	0.099091	(3)	
1000	0.265	0.207547	0.188034	0.188591	(3)	
1100	0.320	0.125000	0.117647	0.117783	(3)	
1200	0.360	0.000000	0.000000	0.000000	(3)	
1300	0.360	-0.027778	-0.028169	-0.028171	(3)	Black Death
1400	0.350	0.214286	0.193548	0.194156	(3)	
1500	0.425	0.282353	0.247423	0.248697	(3)	
1600	0.545	0.128651	0.121833	0.121947	(3)	
1687	0.606	0.495050	0.428266	0.430892	(5)	Plagues of London, Newton's Principia
1750	0.795	0.437736	0.394558	0.395845	(4)	
1800	0.969	0.610939	0.529991	0.533126	(4)	
1850	1.265	0.618182	0.535433	0.538666	(4)	
1900	1.656	0.567633	0.551967	0.552107	(4)	
1910	1.750	0.628571	0.609418	0.609607	(4)	
1920	1.860	1.129032	1.068702	1.069721	(4)	
1930	2.070	1.111111	1.052632	1.053605	(4)	
1940	2.300	1.121739	1.062166	1.063166	(4)	
1950	2.558	1.446443	1.436057	1.436081	(4)	
1951	2.595	1.618497	1.605505	1.605539	(4)	
1952	2.637	1.706485	1.692047	1.692088	(4)	
1953	2.682	1.789709	1.773836	1.773882	(4)	
1954	2.730	1.904762	1.886792	1.886848	(4)	
1955	2.782	1.905104	1.887128	1.887184	(4)	
1956	2.835	1.975309	1.955990	1.956053	(4)	
1957	2.891	1.971636	1.952389	1.952451	(4)	
1958	2.948	1.797829	1.781812	1.781859	(4)	
1959	3.001	1.399533	1.389808	1.389830	(4)	
1960	3.043	1.347355	1.338339	1.338358	(4)	
1961	3.084	1.815824	1.799486	1.799534	(4)	
1962	3.140	2.229299	2.204724	2.204814	(4)	Third discontinuity
1963	3.210	2.211838	2.187644	2.187732	(4)	
1964	3.281	2.103017	2.081134	2.081209	(4)	

1965	3.350	2.089552	2.067947	2.068021	(4)
1966	3.420	2.046784	2.026049	2.026119	(4)
1967	3.490	2.063037	2.041974	2.042045	(4)
1968	3.562	2.105559	2.083623	2.083698	(4)
1969	3.637	2.062139	2.041094	2.041165	(4)
1970	3.712	2.101293	2.079445	2.079520	(4)
1971	3.790	2.005277	1.985371	1.985436	(4)
1972	3.866	1.965856	1.946721	1.946783	(4)
1973	3.942	1.877220	1.859764	1.859817	(4)
1974	4.016	1.817729	1.801357	1.801406	(4)
1975	4.089	1.736366	1.721421	1.721463	(4)
1976	4.160	1.730769	1.715920	1.715962	(4)
1977	4.232	1.701323	1.686973	1.687013	(4)
1978	4.304	1.742565	1.727514	1.727556	(4)
1979	4.379	1.644211	1.630804	1.630840	(4)
1980	4.451	1.864749	1.847524	1.847576	(4)
1981	4.534	1.764446	1.749016	1.749061	(4)
1982	4.614	1.755527	1.740251	1.740295	(4)
1983	4.695	1.682641	1.668603	1.668642	(4)
1984	4.774	1.717637	1.703011	1.703053	(4)
1985	4.856	1.729819	1.714986	1.715028	(4)
1986	4.940	1.740891	1.725868	1.725911	(4)
1987	5.026	1.750895	1.735700	1.735744	(4)
1988	5.114	1.681658	1.667636	1.667675	(4)
1989	5.200	1.692308	1.678108	1.678148	(4)
1990	5.288	1.569592	1.557369	1.557401	(4)
1991	5.371	1.582573	1.570149	1.570181	(4)
1992	5.456	1.502933	1.491723	1.491750	(4)
1993	5.538	1.462622	1.452003	1.452029	(4)
1994	5.619	1.441538	1.431222	1.431246	(4)
1995	5.700	1.403509	1.393728	1.393751	(4)
1996	5.780	1.366782	1.357505	1.357526	(4)
1997	5.859	1.331285	1.322482	1.322501	(4)
1998	5.937	1.296951	1.288595	1.288613	(4)
1999	6.014	1.263718	1.255783	1.255800	(4)
2000	6.090	1.264368	1.256425	1.256441	(4)
2001	6.167	1.248581	1.240835	1.240851	(4)
2002	6.244	1.217168	1.209806	1.209821	(4)
2003	6.320	1.218354	1.210977	1.210992	(4)
2004	6.397	1.203689	1.196488	1.196503	(4)
2005	6.474	1.204819	1.197605	1.197619	(4)
2006	6.552	1.205739	1.198513	1.198528	(4)
2007	6.631	1.191374	1.184319	1.184333	(4)
2008	6.710	1.162444	1.155727	1.155740	(4)
2009	6.788	1.149087	1.142522	1.142535	(4)
2010	6.866				(4)

SUPPORTING INFORMATION

Appendix S4: Data, education and fertility statistics

Contents: Below are the data for Figure 4 in the paper, recorded in Centinel format (1, 2).

Label Fert: Total fertility rate, lifetime births per woman
Label Edu: Average years of total schooling, age 15+, male and female
Label Popul: Population of country, millions
Label Country: Name of country
Label Ref: Source (references follow this appendix)

Table:

Fert	Edu	Popul	Country	Ref
2.2	9.51	40.728738	Argentina	(6)
1.9	11.54	22.323900	Australia	(6)
2.2	5.91	152.862431	Bangladesh	(6)
1.8	10.69	11.047744	Belgium	(6)
3.3	8.25	10.324445	Bolivia	(6)
1.8	7.89	196.935134	Brazil	(6)
2.9	4.72	14.605862	Cambodia	(6)
4.9	6.15	21.156272	Cameroon	(6)
1.6	12.32	34.483975	Canada	(6)
1.8	9.78	17.308449	Chile	(6)
1.7	7.51	1344.130000	China	(6)
2.3	8.95	47.078792	Colombia	(6)
6.1	5.94	63.931512	Congo Dem. Rep.	(6)
4.9	4.65	19.389954	Cote d'Ivoire	(6)
1.4	12.80	10.496088	Czech Republic	(6)
2.6	7.85	10.147598	Dominican Republic	(6)
2.6	7.60	15.246481	Ecuador	(6)
2.8	7.15	79.392466	Egypt	(6)
2.0	10.68	65.371613	France	(6)
1.4	12.37	81.797673	Germany	(6)
4.0	7.00	24.820706	Ghana	(6)
1.4	10.30	11.299976	Greece	(6)
3.9	4.57	14.706578	Guatemala	(6)
3.3	5.11	10.032864	Haiti	(6)
2.5	6.24	1221.156319	India	(6)
2.4	7.61	243.801639	Indonesia	(6)
1.4	9.63	60.723569	Italy	(6)
1.4	11.60	126.740000	Japan	(6)
2.6	11.33	16.558676	Kazakhstan	(6)
4.5	6.14	42.027891	Kenya	(6)
1.2	12.05	49.779000	Korea, South	(6)
2.2	8.79	119.361233	Mexico	(6)
2.7	4.96	32.059424	Morocco	(6)
5.3	1.93	24.581367	Mozambique	(6)
2.5	4.23	27.156367	Nepal	(6)
1.8	11.39	16.693074	Netherlands	(6)
3.3	5.02	176.166353	Pakistan	(6)
2.5	8.88	29.614887	Peru	(6)
3.1	8.43	95.053437	Philippines	(6)
1.3	11.32	38.534157	Poland	(6)
1.4	7.52	10.556999	Portugal	(6)
5.0	2.74	13.330737	Senegal	(6)
1.4	10.27	46.174601	Spain	(6)
2.3	10.06	20.869000	Sri Lanka	(6)
3.0	6.70	21.961676	Syria	(6)
5.4	5.81	46.354607	Tanzania	(6)
1.4	7.99	66.576332	Thailand	(6)
2.1	7.48	10.673800	Tunisia	(6)
2.1	7.05	73.058638	Turkey	(6)
1.5	11.15	45.706100	Ukraine	(6)
2.0	12.24	62.752472	United Kingdom	(6)

1.9	13.18	311.587816	United States	(6)
2.4	8.41	29.500625	Venezuela	(6)
1.8	7.15	87.840000	Vietnam	(6)
4.3	3.68	23.304206	Yemen	(6)
5.8	7.32	13.633796	Zambia	(6)

Supporting references

(1) Lehman C, Williams S, Keen A (2012) The Centinel data format: Reliably communicating through time and place. *Proceedings, International Conference on Information and Knowledge Engineering* IKE2012:47-53. [Link]

(2) Lehman C, Keen A (2013) Using the Centinel data format to decouple data creation from data processing in scientific programs. *Proceedings, International Conference on Scientific Computing* CSC2013:7pp. [Link]

(3) McEvedy C, Jones R (1978). *Atlas of World Population History, Facts on File*, New York, pp. 342–344.

(4) United States Census Bureau: "https://www.census.gov/population"

(5) Interpolated among points of (3) and (4).

(6) World Bank Open Data, including Barro-Lee estimates: "https://data.worldbank.org"

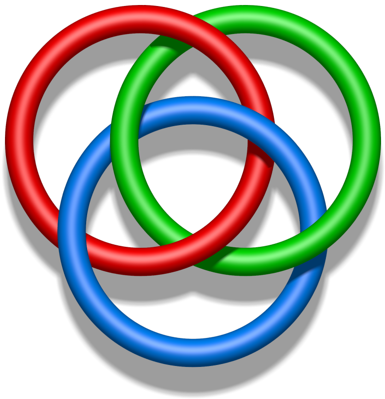

Borromean Rings by Jim Belk, here as art apropos in three-component motifs. No pair of rings actually interlink, yet the three together are unified.
